# Molecular and cellular mechanisms of pulmonary fibrosis

**DOI:** 10.1186/1755-1536-5-11

**Published:** 2012-07-23

**Authors:** Nevins W Todd, Irina G Luzina, Sergei P Atamas

**Affiliations:** 1 Department of Medicine, University of Maryland School of Medicine, Baltimore, MD, USA; 2 VA Medical Center, Baltimore, MD, USA; 3 Division of Pulmonary and Critical Care Medicine, University of Maryland School of Medicine, 110 S. Paca St., Baltimore, MD, 21201, USA

**Keywords:** Idiopathic pulmonary fibrosis, Extracellular matrix, Collagen, Fibroblasts, Epithelial cells, Inflammation, Oxidative stress, Coagulation

## Abstract

Pulmonary fibrosis is a chronic lung disease characterized by excessive accumulation of extracellular matrix (ECM) and remodeling of the lung architecture. Idiopathic pulmonary fibrosis is considered the most common and severe form of the disease, with a median survival of approximately three years and no proven effective therapy. Despite the fact that effective treatments are absent and the precise mechanisms that drive fibrosis in most patients remain incompletely understood, an extensive body of scientific literature regarding pulmonary fibrosis has accumulated over the past 35 years. In this review, we discuss three broad areas which have been explored that may be responsible for the combination of altered lung fibroblasts, loss of alveolar epithelial cells, and excessive accumulation of ECM: inflammation and immune mechanisms, oxidative stress and oxidative signaling, and procoagulant mechanisms. We discuss each of these processes separately to facilitate clarity, but certainly significant interplay will occur amongst these pathways in patients with this disease.

## Review

### Introduction

Pulmonary fibrosis is a chronic lung disease characterized pathologically by excessive accumulation of extracellular matrix (ECM) and remodeling of the lung architecture, and additionally characterized by recognizable clinical, physiologic, and radiographic findings. Though some descriptions of fibrous diseases of the lungs can be found as early as the 5th century BC by Hippocrates [1,2], more modern descriptions of pulmonary fibrosis occurred in the early part of the 20th century with reports by Hamman and Rich of four patients with rapidly progressive diffuse interstitial fibrosis of the lungs [3,4]. Although the prognosis of patients with diffuse pulmonary fibrosis is poor, it was subsequently realized that many patients did not have the extremely rapid deteriorating course that was described by Hamman and Rich. With further pathologic analysis, several distinct types of pulmonary fibrosis were described, and the terms diffuse fibrosing alveolitis, diffuse interstitial fibrosis, and idiopathic pulmonary fibrosis (IPF) were introduced to describe a more insidious, yet still debilitating form of chronic pulmonary fibrosis [5,6]. Currently, IPF is considered the most common and severe form of pulmonary fibrosis, with a disheartening median survival of approximately three years, with no proven effective therapy, and with lung transplantation remaining the only viable intervention in end-stage disease [7].

The pathologic findings in pulmonary fibrosis (excessive accumulation of ECM and remodeling of the lung architecture) are a consequence of disturbances in two physiologically balanced processes: proliferation and apoptosis of fibroblasts, and accumulation and breakdown of ECM. When the normal balance between ECM deposition and turnover is shifted toward deposition or away from breakdown, excessive ECM accumulates. When the balance between fibroblast proliferation and apoptosis is shifted toward accelerated proliferation or slowed apoptosis, fibroblasts - the primary ECM producers - accumulate. Several possible origins of ECM-producing mesenchymal cells have been described, and have included accumulation of resident lung fibroblasts, homing and fibroblastic differentiation of bone marrow-derived cells such as circulating fibrocytes or monocytes [8-11], or epithelial-mesenchymal transition (EMT) [12]. Independent of the source of fibroblast expansion in the lungs (resident or systemic), it seems agreed upon that the ultimate effector cell in pulmonary fibrosis is the myofibroblast, a differentiated fibroblast which has contractile properties similar to smooth muscle cells, and which is characterized by the presence of alpha-smooth muscle actin (α-SMA).

In addition to altered mesenchymal cells, abnormalities of the alveolar epithelium in patients with pulmonary fibrosis have been noted from the earliest descriptions of the disease process [13,14]. Loss of normal type I alveolar epithelium and replacement by hyperplastic type II cells or bronchiolar cuboidal cells is a consistent finding in patients with IPF. In addition to these observations, more recent mechanistic studies have focused on the interplay, or cross-talk, between damaged epithelial cells and lung mesenchymal cells. This epithelial-mesenchymal interplay lends support to a key theme in pulmonary fibrosis, in which altered lung mesenchymal cells coupled with alveolar epithelial cell injury result in the accumulation of ECM and remodeling of the lung architecture.

**Figure 1:**
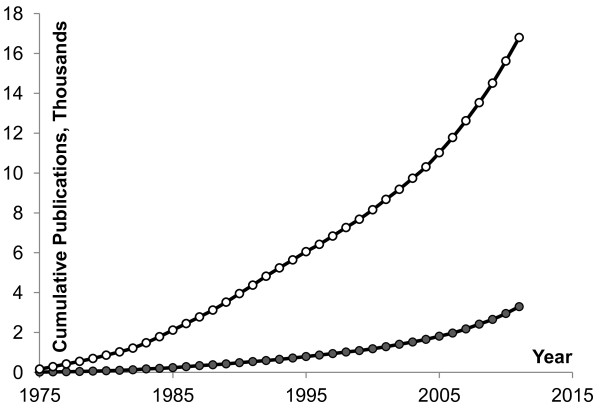
**Cumulative number of publications using PubMed searches for articles on pulmonary fibrosis, excluding cystic fibrosis.** Search strategy (open circles) was ‘((lung OR pulmonary) AND (fibrosis OR fibrotic) AND english [la] AND hasabstract) NOT cystic’, or when referred to in the title, search strategy (closed circles) was ‘((lung [ti] OR pulmonary [ti]) AND (fibrosis [ti] OR fibrotic [ti]) AND english [la] AND hasabstract) NOT cystic [ti]’. These cumulative numbers most likely underestimate the realistic breadth of literature on the topic, as pulmonary fibrosis is often described using various terms (fibrosing alveolitis, interstitial lung disease), and a large body of literature has focused on cellular and molecular responses in cell culture (fibroblasts, epithelial, endothelial, and inflammatory cells) without mentioning pulmonary fibrosis in the title or the abstract.

**Figure 2:**
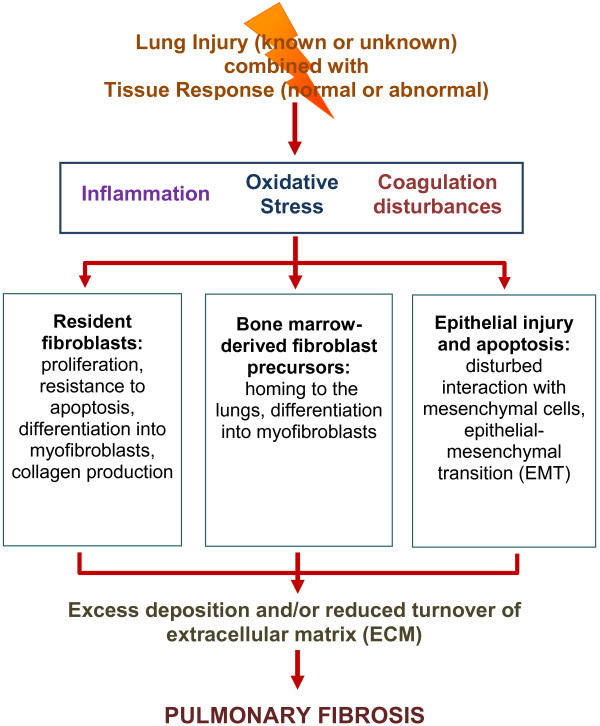
Schematic diagram representing three broad mechanisms (inflammation, oxidative stress, and coagulation disturbances) that alone or in combination may be responsible for alterations in mesenchymal cells, epithelial cells, and extracellular matrix (ECM) that result in pulmonary fibrosis following lung injury.

An extensive body of scientific literature regarding pulmonary fibrosis has accumulated over the past 35 years, and Figure 1 shows the accelerating pace of research in this field over the past two decades. Although the precise mechanisms that drive the development of fibrosis in most patients remain incompletely understood, three broad areas (as seen in Figure 2) have been explored that may be responsible for the combination of altered lung fibroblasts, loss of alveolar epithelial cells, and excessive accumulation of ECM: (1) inflammation and immune mechanisms: the role of acute and chronic inflammation driven by cytokines, cells or cell surface molecules; (2) oxidative stress and oxidative signaling: the role of reactive oxygen species; and (3) a procoagulant milieu in the lung: the role of the coagulation proteinases and their tissue receptors. Each of these processes will be considered separately to facilitate clarity, but we hope the reader will appreciate the significant interplay that may occur amongst these pathways in patients with pulmonary fibrosis.

### Inflammation and immune mechanisms

From the earliest descriptions of patients with pulmonary fibrosis, cellular inflammation in the lung parenchyma has been a consistent pathologic finding [3-6]. Histologic analysis has shown varied accumulations of lymphocytes, macrophages, plasma cells, eosinophils and neutrophils, and the presence of lymphoid follicles with germinal centers has been observed in many patients in the lung interstitium [5,6]. The initial terms ‘diffuse fibrosing alveolitis’ and ‘cryptogenic fibrosing alveolitis’ were used as a reflection of the inflammatory component of the pathologic process in pulmonary fibrosis [5,15], and most patients with usual interstitial pneumonia (UIP), the pathologic hallmark of IPF, will manifest a mild to moderate degree of chronic cellular inflammation in the lung [7,16-18].

Over the past 15 to 20 years, however, the role of inflammation has been questioned, and the hypothesis has been put forward that active cellular lung inflammation is not a major feature or requirement for the development of IPF. Recent pathologic descriptions of patients with UIP have emphasized the epithelial, mesenchymal, and ECM abnormalities while de-emphasizing the cellular inflammatory features. This paradigm has thus shifted emphasis in IPF towards a process of epithelial cell injury coupled with exaggerated wound healing, and inflammation has been relegated to a mechanistically less important and bystander role in the fibrotic process [19,20]. Probably the major supporting argument for inflammation playing a non-causal role stems from the observation that anti-inflammatory therapies, particularly corticosteroids, have been uniformly ineffective in improving pulmonary function or survival in patients with IPF [21,22].

However, even though the role of inflammation in IPF has been recently de-emphasized, the original findings of cellular inflammation in the lung have been supplemented with an extensive accumulation of scientific studies which have implicated numerous inflammation-related cytokines and cell surface molecules in profibrotic mechanisms. It is also important to note that resistance to corticosteroids does not necessarily indicate or equate with a lack of inflammatory involvement, as there are several well-established diseases in which inflammation is clearly accepted to be the predominant underlying mechanism, but in which traditional anti-inflammatory therapy has been poorly effective [23]. Stemming from the observations on inflammatory cells, cytokines, chemokines, and cell surface molecules, the inflammation hypothesis has dominated the field of pulmonary fibrosis for nearly four decades, and IPF continues to be viewed by many authorities as a chronic inflammatory disease of the lung parenchyma [23-26].

It is difficult if not impossible to comprehensively review the evidence in support of the role of inflammation in pulmonary fibrosis. Such evidence is abundant from mechanistic studies in cell culture, experimental research in animals, and observations in human patients. Below, we provide an overview of such evidence and lines of thought, in hopes to provide a springboard for continued independent studies in the field.

### Role of cytokines

Over the past 40 years of research into mechanisms of pulmonary fibrosis, an immense amount of literature has described alterations in cytokine expression and function in animals and patients with pulmonary fibrosis. Most of the findings have described a propensity for a variety of cytokines to promote and enhance the fibrotic process, whereas in some instances, up-regulation of a particular cytokine is associated with inhibition of fibrosis. In Table 1, we have listed the cytokines which we currently interpret as most relevant to mechanisms of pulmonary fibrosis, and have summarized the most pertinent *in vitro*, animal model, and human trial data for each cytokine. The potential sources of these cytokines in the lung are numerous, and include resident or systemic epithelial, mesenchymal, or inflammatory cells (T lymphocytes, B lymphocytes, macrophages, neutrophils, eosinophils, and platelets).

**Table 1:** Cytokines involved in the regulation of pulmonary fibrosis

**Mediators**	**Effects Relevant to Pulmonary Fibrosis**	**References**
**Growth Factors**		
Transforming growth factor-β (TGF-β)	The major profibrotic growth factor. In vitro*,* stimulates fibroblast ECM production, myofibroblast differentiation, resistance to apoptosis, and production of ROS. Induces apoptosis in epithelial cells, and promotes epithelial-mesenchymal transdifferentiation (EMT). Upregulated in animal models of fibrosis, and overexpression in vivo induces severe fibrosis.	[12,27-36]
Connective tissue growth factor (CTGF, CCN2)	Stimulates fibroblast proliferation and ECM production in vitro. Upregulated in the bleomycin model, and overexpression in vivo induces mild fibrosis. Functions in combination with TGF-β.	[37-44]
Platelet-derived growth factor (PDGF)	Stimulates fibroblast proliferation and chemotaxis in vitro. Upregulated in animal models of fibrosis, and inhibition reduces fibrosis. Upregulated in human fibrotic diseases, but inhibition did not improve survival in patients with IPF.	[38,44-54]
Insulin-like growth factor (IGF)	Stimulates fibroblast ECM production in vitro. Upregulated in the bleomycin model, but overexpression in vivo did not induce fibrosis. Stimulates proliferation of epithelial cells.	[44,55-59]
Interleukin-4 (IL-4)	Th-2 cytokines which stimulate fibroblast proliferation and ECM production in vitro. Upregulated in the bleomycin model, and overexpression in vivo induces fibrosis. Induce alternative activation of macrophages.	[24,25,60-68]
Interleukin-13 (IL-13)		
Interferon-γ (IFN-γ)	Pro-inflammatory Th-1 cytokine which inhibits fibroblast proliferation and ECM production in vitro, and enhances fibroblast apoptosis. In vivo, reduces fibrosis in the bleomycin model, but administration in patients with IPF did not improve survival.	[69-77]
Interleukin-1β (IL-1β)	Pro-inflammatory cytokines which in vitro stimulate fibroblast proliferation and chemotaxis, but inhibit collagen production. Upregulated in the bleomycin model, and overexpression in vivo induces inflammation and fibrosis, with fibrosis likely mediated by TGF-β. Inhibition of TNF-α in patients with IPF did not improve outcomes.	[44,78-88]
Tumor necrosis factor-α (TNF-α)		
Interleukin-17 (IL-17)	Pro-inflammatory cytokine which is upregulated in the bleomycin model. Exogenous administration in vivo induced fibrosis, which was reduced by blockade of TGF-β. Upregulated in patients with IPF.	[25,89,90]
Oncostatin M (OSM)	In fibroblasts, stimulates proliferation and ECM production in vitro, and inhibits apoptosis. Overexpression and exogenous administration in vivo induces inflammation and fibrosis, and fibrosis occurred independently of TGF-β.	[91-96]
Interleukin-10 (IL-10)	Anti-inflammatory cytokine which inhibits fibroblast ECM production in vitro*,* and upregulation or overexpression in the bleomycin model reduced fibrosis. In vivo overexpression alone, however, induced fibrosis, likely mediated by CCL2. Induces alternative activation of macrophages.	[68,97-100]
**Chemokines**		
CCL2 (MCP-1)	Pro-inflammatory CC chemokine which stimulates fibroblast ECM production and resistance to apoptosis in vitro. Upregulated in animal models of fibrosis, and in vivo loss of function (chemokine or receptor) reduces fibrosis. Recruits bone-marrow derived fibrocytes to the lung.	[98,101-108]
CCL18 (PARC)	Pro-inflammatory CC chemokine which mildly stimulates fibroblast ECM production in vitro*.* Overexpression in vivo induces fibrosis, however, overexpression combined with bleomycin reduced fibrosis. Serum concentrations inversely correlated with outcomes in patients with IPF and SSc.	[109-115]
CCL3 (MIP-1α)	Pro-inflammatory CC chemokine which is upregulated in animal models of fibrosis, and in vivo loss of function (chemokine or receptor) reduces fibrosis. Recruits bone-marrow derived fibrocytes to the lung.	[116-119]
CXCL12	CXC chemokine which is upregulated in the bleomycin model, and is the major chemokines responsible for recruiting bone-marrow derived fibrocytes to the lung. Upregulated in BAL and serum in patients with IPF, and inversely correlated with physiologic parameters.	[120-122]

There are additional cytokines (for example, TGF-α, IFN-α, fibroblastic growth factor, IL-6) not included in Table 1 which have been studied in pulmonary fibrosis, but in our judgement, their regulatory role in fibrosis has been less clearly defined, and the high level of complexity of their regulation does not allow for clear designation as profibrotic or antifibrotic. Additionally, many small molecules (for example, serotonin, endothelin, leptin, lysophosphatidic acid, histamine, angiotensin) have also been studied in pulmonary fibrosis, but likewise their roles in fibrosis are less clearly defined at present.

### Role of inflammatory cells

#### T lymphocytes

A consistent finding in most fibrotic diseases of the lungs is the presence of T lymphocytes, one of the major inflammatory cell types. Mechanistic studies regarding the role of T cells in pulmonary fibrosis have been performed in animals which lacked T lymphocytes or had selective attraction of T lymphocytes to the lungs. Most of these studies used bleomycin as a model of lung injury, in which fibrosis is preceded by pulmonary inflammation.

In athymic mice lacking T cells, administration of bleomycin resulted in less fibroblast proliferation and less ECM accumulation compared to wild-type mice [123]. Mice whose T lymphocytes lacked CD28, a central co-stimulatory cell surface molecule necessary for full T cell activation, showed markedly attenuated fibrosis following exposure to bleomycin, and transferring CD28-positive T lymphocytes into these CD28-deficient animals restored the fibrotic response to bleomycin [124]. In mice systemically depleted of T lymphocytes, exposure to bleomycin resulted in less collagen accumulation and increased survival compared with controls [125,126]. In our mouse model in which selective attraction of T lymphocytes to the lung was achieved by over-expressing human CCL18 (PARC), a prolonged infiltration of T cells occurred, moderate collagen accumulation developed, and systemic depletion of T cells prevented the collagen accumulation despite the continuous expression of CCL18, suggesting that T cells were indeed the driving force of fibrosis [113]. It should be noted that although human CCL18 appears to be fully functional in mice in cell culture and in vivo [109-113, ][127], the chemokine CCL18 has not been definitely identified in mice.

There may be a complex interplay among subtypes of T lymphocytes in pulmonary fibrosis, particularly between T effector and T regulatory cells. T regulatory lymphocytes (T regs) are profibrotic and immunosuppressive, and exert their profibrotic actions primarily via transforming growth factor-beta (TGF-β) and platelet-derived growth factor (PDGF) [128]. In an animal model of silica-induced pulmonary fibrosis, T regs were recruited to the lungs, caused fibroblast proliferation, had increased expression of TGF-β1 and PDGF, and caused pulmonary fibrosis upon transfer into silica-unexposed animals [45]. In silica-exposed animals depleted of T regs, pulmonary fibrosis occurred, but increased numbers of CD4+ T effectors were present. These T effectors caused fibroblast proliferation, caused pulmonary fibrosis upon transfer into T reg deficient (but not T reg competent) animals, and neutralizing antibody against these T effectors eliminated collagen accumulation [45]. These results suggest a complex interplay between lymphocyte subsets, with T regulatory cells themselves acting profibrotically, and the role of CD4+ T effector cells depending on the presence or absence of T regulatory cell competence.

Observational studies in humans link T cell infiltration to fibrosis. In patients with IPF, the presence of T cells within lung tissue and BAL of patients has been consistently observed [129-134]. These T cells are activated and antigen experienced [132,135,136], and characterization has shown that both CD4+ and CD8+ cells are present, with the suggestion that CD8+ T cells represent the majority [129-132,137]. Increased numbers of CD8+ T cells in lung tissue from IPF patients was associated with worse pulmonary outcomes [130], and in BAL fluid, higher CD4/CD8 ratios correlated with an improved clinical response to anti-inflammatory therapy [133]. In patients with IPF, well-organized lymph-node-like structures with features of ‘lymphoid neogenesis’ are present, which are composed of T cells expressing CD40L, B cells, and mature dendritic cells [132]. These T and B cells are activated, but not proliferating, and most are CD45RO + indicative of a memory phenotype [132]. These findings support the concept that organized lymphoid tissue can persist in the lung and contribute to chronic inflammation even in the absence of cellular proliferation.

In other forms of interstitial pneumonia, accumulation of T cells has been shown in BAL from patients with scleroderma [138,139], rheumatoid arthritis [140], and polymyositis/dermatomyositis [141,142]. In general, it appears that although both CD4+ and CD8+ T cells are associated with pulmonary fibrosis, CD8+ T cells appear to be associated with a worse prognosis. In patients with sarcoidosis [143], beryliosis [144], rheumatoid arthritis [140], and hypersensitivity pneumonitis [145], CD4+ T cells were predominant in the lungs, consistent with the better prognosis of these diseases compared with IPF.

Thus, there are numerous observations in animal models and in patients which describe an association of pulmonary fibrosis with T lymphocytic infiltration. The preponderance of evidence suggests that such association is reflective of T cell profibrotic action, but depending on cell phenotype and the nature of the pulmonary milieu, T cells may promote or diminish the pulmonary fibrotic process. Two broad mechanisms by which T cells may influence the fibrotic process include production of Th-1 or Th-2 cytokines (as seen in Table 1) and cell surface molecule interactions with epithelial or mesenchymal cells, which will be described subsequently below.

#### Macrophages

Macrophages constitute the majority of cells recovered from BAL of normal individuals, and have essential roles in phagocytosis, innate and adaptive immunity, and surfactant homeostasis [146]. A pathogenic role for macrophages in pulmonary fibrosis has been postulated for many years, and described mechanisms have included overexpression of reactive oxygen species [147-151], proteinase-activated receptors [152-154], Fas ligand [155], and profibrotic cytokines [97,154,156-162].

One feature regarding a potential role of macrophages in pulmonary fibrosis relates to the concept of alternative activation. Classical activation of macrophages is induced by interferon-γ, and is characterized by macrophage expression of IL-1β, IL-6, TNF-α, and nitric oxide. Alternative activation of macrophages is induced most notably by the Th-2 cytokines IL-4 and IL-13, and is characterized by macrophage expression of mannose receptor-1 (CD206), arginase-1, the lectin-binding protein Ym1, the resistin-like protein Fizz1, and the chemokine CCL18 [60,161,162].

Several animal models of pulmonary fibrosis have demonstrated that alveolar macrophages (AM) display a phenotype consistent with alternative activation. In the silica-induced model, mRNA expression of Ym1 in the lung increased significantly in wild-type mice consistent with alternative activation, but did not increase in silica-exposed IL-4R null mice, indicating that a Th-2 milieu was necessary for alternative activation and fibrosis [162]. In the herpes virus-induced and transgenic TGF-β models of fibrosis, AM accumulated in the lungs and expressed the alternative activation markers Ym1/2, Fizz1 and arginase-1 [163-165].

Likewise, in patients with pulmonary fibrosis, the preponderance of data suggests that AM display an alternatively activated phenotype. In patients with IPF compared to healthy controls, AM showed increased expression of CD206, and spontaneously generated higher levels of the proinflammatory cytokines CCL17, CCL18, and CCL22 [97,161]. Co-culture of cell supernatants from alternatively activated AM of IPF patients with normal lung fibroblasts generated higher amounts of collagen production than supernatants from AM of healthy controls [161]. In patients with IPF, expression of arginase-1 was increased compared with healthy controls, as demonstrated by immunostaining of AM, immunostaining of lung tissue in areas of interstitial fibrosis, and by higher levels of arginase-1 protein in lung tissue lysates [163].

The above constellation of findings indicate that alternative activation of macrophages may play a mechanistic role in pulmonary fibrosis. Blocking alternative macrophage activation may prove therapeutic, and development of such new approaches to therapies for pulmonary fibrosis is under way. For example, serum amyloid P inhibited bleomycin and TGF-β induced pulmonary fibrosis at least partially through attenuation of alternative macrophage accumulation [164,165].

#### B lymphocytes

Though most literature regarding potential lymphocyte involvement in mechanisms of fibrosis has referenced T cells, a role for B cells has been suggested for many years. As mentioned previously, patients with IPF often have well-organized lymph-node-like structures in the lung composed of activated T and B cells [132]. Production of antibody is a major function of B cells, and production of autoantibodies has been demonstrated against alveolar epithelial cell antigens such as vimentin and cytokeratins in patients with IPF [166-171]. Most recently, autoantibodies against periplakin, a component of desmosomes within epithelium, were identified in patients with IPF, and were associated with worse physiologic parameters [172].

A mechanistic role for B cells in pulmonary fibrosis is also supported by animal and patient data regarding CD19, a crucial cell-surface signaling molecule which is expressed on B cells and regulates B cell function. In patients with systemic sclerosis (SSc), an autoimmune disease in which pulmonary fibrosis is a major cause of morbidity and mortality, systemic B cells overexpress CD19 compared to healthy controls, and polymorphisms in CD19 are associated with an increased susceptibility to SSc [173,174]. In an animal model of SSc, mice deficient in CD19 showed attenuated pulmonary and dermal fibrosis in response to subcutaneous bleomycin compared with wild-type controls [175]. In the intratracheal bleomycin model, overexpression of CD19 correlated with increased histologic lung fibrosis, increased lung hydroxyproline content, and reduced survival compared to wild-type controls, and mice deficient in CD19 had reduced fibrosis and improved survival compared to controls [176]. The number of B cells in BAL correlated with CD19 expression, and CD19 expression was necessary for B cell accumulation [176]. Since it has been demonstrated that the presence of CD19 alters B cell phenotype, the above results suggest that CD19 may have a mechanistic role in fibrosis by skewing B cells towards profibrotic cytokine expression.

Conversely, the presence of B cells in fibrosis may be protective. In the silica-induced model, overexpression of the anti-inflammatory cytokine IL-9 was accompanied by an expansion of B cells within the lungs and with reduced lung fibrosis [177,178]. The presence of B cells was necessary for the protective effect of IL-9, demonstrated by lack of reduction in fibrosis in B cell-deficient mice, and by a restoration of the reduction in fibrosis by B cell reconstitution [178]. The concept of conflicting roles for B cells in fibrosis is similar to that postulated for T cells, in which depending on cell phenotype and the nature of the pulmonary milieu, lymphocytes may promote or diminish the pulmonary fibrotic process.

#### Fibrocytes

Bone-marrow-derived cells that produce collagen and express markers of leukocytes (CD45) and stem cells (CD34) normally circulate at low frequencies, below 1% of circulating cells. These cells were termed fibrocytes in a seminal study [9]. In response to local and systemic injuries, including pulmonary insults, fibrocytes are released from bone marrow in higher numbers, and home to sites of injury, where they differentiate into myofibroblasts and contribute to ECM deposition [8,9,120,121]. Additionally, circulating monocytes may home to sites of injury and differentiate into mesenchymal cells [10,11]. Recruitment of bone-marrow-derived mesenchymal progenitors is driven by several cytokines that are produced by injured tissues, including CXCL12, CCL2, CCL3, and IL-10 [98,101,116,122]. These observations indicate that anti-fibrotic therapies should be limited not only to the organ affected by the disease, but have systemic mechanisms of action as well.

### Role of cell surface molecules

#### CD40-CD40L

CD40 ligand (CD40L or CD154) and CD40, members of the TNF family of cytokines and receptors, respectively, are cell surface molecules first identified as co-stimulatory molecules involved in the process of immune cell activation, with activated CD4+ T cells expressing CD40L, and activated antigen presenting cells expressing CD40. It was subsequently demonstrated that CD40 may also be expressed on several non-hematopoietic cell types [179], including human lung fibroblasts [180], which raised the possibility that CD40/CD40L interactions may affect fibroblast function. Subsequent work showed that engagement of CD40 on fibroblasts by CD40L or by agonistic antibody caused cell proliferation [61,181], mobilization of NF-κB [179,182], production of IL-6 and IL-8 [179,181,182], and expression of ICAM-1 and VCAM-1 [181]. Combined stimulation with IL-4, a potent stimulant for fibroblast proliferation, and ligation of CD40 had synergistic effects on enhancing fibroblast proliferation [61]. In animals, disruption of the engagement of CD40 by antibody against CD40L protected against radiation-induced, oxygen-induced, and autoimmune models of pulmonary injury and fibrosis [183-185].

In patients with IPF, areas of lymphocyte aggregates are often present which contain large numbers of activated T cells expressing CD40L [132]. Additional sources of CD40L include platelets, which are present in areas of lung injury due to the increased procoagulant milieu, and dendritic cells, which are present in lymphocyte aggregates in patients with IPF [132,186,187]. Interestingly, CD40L expression was also found in primary human fibroblasts, and found to be expressed and elevated in fibroblasts from patients with IPF compared to normal controls [188].

The constellation of findings suggests that the CD40/CD40L system may be an important pathway by which several cell types rich in CD40L may promote and perpetuate fibrosis through engagement of CD40 on fibroblasts [182,189]. The finding that lung fibroblasts also express CD40L suggests that fibroblasts themselves may be able to perpetuate fibrosis in an autocrine and paracrine fashion via the CD40/CD40L pathway [188].

#### Fas-FasL

Fas (Fas antigen, Fas receptor, or CD95) is a member of the TNF family of cell surface receptors, is expressed in numerous cell types, and induces cellular apoptosis once engaged by FasL. FasL (Fas ligand or CD178) is a transmembrane protein belonging to the TNF family, is predominantly expressed on activated T lymphocytes and natural killer cells, and induces apoptosis in Fas-bearing cells. The role of the Fas-FasL pathway in pulmonary fibrosis has been examined in both epithelial and mesenchymal cells.

Bronchiolar and alveolar epithelial cell apoptosis has been a consistent finding in the bleomycin model, along with up-regulation of Fas mRNA and Fas pathway genes in epithelial cells, and up-regulation of FasL in lung tissue and infiltrating lymphocytes [190,191]. In mice, inhalation of agonistic anti-Fas antibody alone caused apoptosis of bronchiolar and alveolar epithelial cells, increased inflammation in BAL and lung tissue, increased collagen content, and increased lung tissue TGF-β mRNA similar to that observed with bleomycin [192,193]. Inhalation or injection of soluble Fas (aimed at binding and neutralizing inherent FasL) along with bleomycin reduced epithelial cell apoptosis, tissue inflammatory cell infiltration, and collagen accumulation [194]. Mice deficient in Fas (*lpr*) or FasL (*gld*) had substantially reduced tissue inflammatory cells, epithelial cell apoptosis, and collagen accumulation compared to controls in response to bleomycin challenge [191,194]. Conversely, selective inactivation of Fas in T lymphocytes (via Cre-mediated recombination) led to up-regulation of T cell FasL, massive infiltration of inflammatory cells in the lungs, and development of pulmonary fibrosis [195]. Treatment with neutralizing anti-FasL antibody completely prevented the accumulation of lymphocytes in the lung [195].

In mesenchymal cells, the preponderance of mechanistic data suggests inherent resistance to Fas-mediated apoptosis. In primary lung fibroblasts, ligation of cell surface Fas by agonistic antibody was unable to induce apoptosis, and resulted in increased levels of the anti-apoptotic proteins X-linked inhibitor of apoptosis (ILP) and FLICE-like inhibitor protein (FLIP_L_) [27]. Similarly, fibroblasts from patients with pulmonary fibrosis demonstrated resistance to apoptosis when exposed to recombinant FasL, and demonstrated prominent signals for ILP and FLIP_L_ in lung tissue [27,196]. Despite inherent resistance to Fas-mediated apoptosis, several studies have shown that TNF-α and IFN-γ, particularly in combination, increase Fas expression in fibroblasts and increase susceptibility to apoptosis [27,69,196,197]. Attenuation of Fas expression in fibroblasts by small interfering RNA (siRNA) inhibited the ability of TNF-α and IFN-γ to increase susceptibility to apoptosis, whereas transduction of fibroblasts with Fas-expressing adenovirus-enhanced apoptosis when engaged with agonistic antibody [69]. The ability of TNF-α and IFN-γ to sensitize fibroblasts to apoptosis suggests that altered pro-inflammatory cytokine milieus may contribute to the development of pulmonary fibrosis.

It appears that enhanced apoptosis in epithelial cells coupled with resistance to apoptosis in mesenchymal cells affects the cross-talk between these two cell types. This altered cross-talk is often mediated by TGF-β. In the bleomycin model, myofibroblasts demonstrated overexpression of FasL and induced epithelial cell apoptosis in vitro [191]. In epithelial cells, TGF-β induced apoptosis in vitro, and low concentrations of TGF-β, which were unable to induce epithelial cell apoptosis alone, increased apoptosis in epithelial cells stimulated with agonistic anti-Fas antibody or soluble FasL [28]. In vivo administration of TGF-β along with agonistic anti-Fas antibody increased epithelial cell apoptosis to a degree greater than with either agent alone, and the induction of epithelial cell apoptosis by soluble FasL was inhibited by antibodies against TGF-β [28].

Observational studies in patients with IPF have demonstrated up-regulation of Fas and Fas-signaling molecules in epithelial cells compared to normal controls [69,198,199]. Mesenchymal cells within fibroblastic foci demonstrated minimal or absent expression of Fas, and fibroblasts from patients with pulmonary fibrosis had lower expression of surface bound Fas, but higher levels of soluble Fas in the supernatant [196]. In regards to FasL, there was increased expression of soluble FasL in BAL and serum in patients with active IPF and connective tissue disease-interstitial pneumonia [198,200,201]. In lung tissue from patients with IPF, increased FasL protein was demonstrated in infiltrating granulocytes and T cells, and strong expression of FasL was seen in myofibroblasts [191,198]. In non-smoking patients with IPF, BAL showed increased percentages of alveolar macrophages and CD8+ cells expressing FasL along with increased levels of soluble FasL, and these findings were inversely correlated with vital capacity [155]. The summative observations in cell culture, animal models, and patients support a mechanistic role for disturbances in Fas and FasL in the development of pulmonary fibrosis.

#### Integrins

Integrins are heterodimeric, transmembrane cell surface molecules which primarily mediate cell-cell and cell-ECM adhesion, but also play major roles in cell migration, growth, and survival [202-205]. Currently, eighteen α-subunits and eight β-subunits have been identified which associate to form 24 known integrins. Mechanistic roles for integrins in the pathogenesis of pulmonary fibrosis have been described in epithelial, inflammatory, and mesenchymal cells.

The integrin αvβ6, which is expressed principally in epithelial cells, is one of the αv-integrins which has the ability to activate latent TGF-β by binding to the tripeptide RGD sequence on TGF-β latency-associated peptide (LAP). In a landmark study, αvβ6 activated latent TGF-β by binding to the RGD sequence, and in the bleomycin model, mice lacking β6 developed increased pulmonary inflammation, but were protected from pulmonary fibrosis [206]. In the animal model of radiation-induced fibrosis, β6 was up-regulated following injury, and lack of β6 or mutation of integrin binding site on TGF-β LAP significantly reduced pulmonary fibrosis [207]. In the radiation-induced or bleomycin model, antibody against αvβ6 reduced histologic evidence of fibrosis, hydroxyproline content, and phosphorylation of nuclear Smad 2/3 [207,208]. In patients with IPF, αvβ6 was overexpressed within alveolar epithelial cells compared with normal controls, and in patients with systemic sclerosis, was overexpressed to a greater extent in patients with a UIP *vs.* NSIP pathologic pattern [208].

The integrin αvβ8 is also expressed in epithelial cells and fibroblasts. In fibroblasts, αvβ8 contributes to TGF-β activation, fibrosis, and regulation of immune processes including dendritic cell function [209]. In airway epithelial cells, the β8 subunit was highly expressed, active TGF-β was produced, and airway proliferation was minimal [210]. Antibody against β8 or TGF-β reduced active TGF-β production and resulted in enhanced airway proliferation, indicating that β8 activation of latent TGF-β was regulating epithelial cell proliferation. In an epithelial wounding model, administration of TGF-β delayed wound closure, and antibody against αvβ8 reduced activation of latent TGF-β and enhanced epithelial wound closure [211]. Both of these studies suggest that activation of latent TGF-β by αvβ8 may contribute to the broad mechanism of impaired epithelial cell regeneration coupled with mesenchymal cell proliferation in patients with pulmonary fibrosis.

The integrin α3β1 is an epithelial cell integrin and laminin receptor. Specific loss of α3 expression in lung epithelial cells of mice exposed to bleomycin resulted in typical findings of acute inflammation and lung injury, but had reduced accumulation of myofibroblasts and type I collagen [212]. Specific loss of α3 expression resulted in an inability to form β-catenin/Smad2 complexes, a process implicated in the development of epithelial-mesenchymal transition (EMT), suggesting that the α3β1 integrin may play a central role in EMT [212,213].

Several integrins have been examined in inflammatory cells in pulmonary fibrosis. First, in our animal model of CCL18 overexpression, T lymphocytes accumulated in the lungs, expressed αvβ3 and αvβ5 integrins, and administration of neutralizing antibody against αv or genetic deficiency of β3 significantly reduced pulmonary T cell infiltration and collagen accumulation [214]. Transformed T cells that overexpressed αvβ3 and αvβ5 stimulated collagen accumulation in co-cultured fibroblasts, which was mediated by TGF-β, and pulmonary T cells from patients with systemic sclerosis expressed αvβ3 and αvβ5 integrins [214]. Second, many T cells express αEβ7, which is up-regulated by TGF-β, and binds to E-cadherin on epithelial cells [215]. In the bleomycin model, the majority of CD8+ and γδ T cells in BAL expressed αEβ7 [216], and in patients with IPF, a significantly higher percentage of CD4+ and CD8+ T cells in BAL expressed αEβ7 when compared to peripheral blood [217]. Third, lymphocytes and eosinophils may express α4 integrin, which binds to vascular cell adhesion molecule-1 (VCAM-1) on endothelium [218]. In the bleomycin model, treatment with neutralizing antibody against α4 resulted in reduced cellular inflammation, lipid peroxidation, hydroxyproline content, histologic fibrosis, and α-SMA compared with controls [219]. Though the precise role of T cells in the pathogenesis of pulmonary fibrosis remains incompletely understood, T cell expression of several integrins which bind epithelium or ECM skew the pulmonary milieu towards a pro- or anti-phenotype depending on the T cell phenotype.

Integrins expressed on fibroblasts have been shown to activate latent TGF-β and promote fibrosis. The α2β1 integrin is a major type I collagen receptor [220]. In normal fibroblasts, exposure to polymerized collagen inhibited proliferation, whereas fibroblasts from patients with IPF demonstrated abnormal proliferation due to activation of the PI3K-Akt-S6K1 pathway and low activity of the tumor suppressor phosphatase and tensin homologue (PTEN) [221]. Protein expression of PTEN was preserved, but there was defective regulation of PTEN function by the α2β1-polymerized collagen interaction. Consistent with these in-vitro experiments, mice haploinsufficient for PTEN showed an exaggerated fibroproliferative response and increased collagen deposition in a cutaneous and bleomycin injury model, and immunohistochemistry in patients with IPF showed activation of Akt in fibroblastic foci [221].

Myofibroblast contraction has been shown to activate TGF-β by inducing conformational changes in LAP mediated by integrin binding. Contraction of myofibroblasts along with mechanically-resistant ECM activated latent TGF-β via αvβ5 binding, and blocking antibodies against αvβ5 prevented this TGF-β activation [222]. Thy-1, a cell surface glycoprotein which inhibits a fibrogenic phenotype in fibroblasts and is associated with decreased fibrosis in the bleomycin model [223,224], may have a role in cell contraction-mediated TGF-β activation. Several studies have shown that Thy-1 can bind to integrins, and specifically, Thy-1 bound αvβ5 in a cell-free system and on the surface of lung fibroblasts [225]. Upon exposure to cell contraction agonists, fibroblasts either lacking Thy-1 or in which Thy-1/αvβ5 binding was prevented were able to activate latent TGF-β and promote myofibroblast differentiation, whereas these effects were absent in fibroblasts expressing Thy-1 [225]. Thesis data suggested that Thy-1 is able to bind αvβ5 on the cell surface, and prevent myofibroblast contraction-induced integrin-dependent activation of latent TGF-β.

In patients with localized or diffuse scleroderma, fibroblasts demonstrated increased expression of αvβ5, and exposure to exogenous latent TGF-β increased collagen production [226,227]. Overexpression of αvβ5 on normal fibroblasts recruited latent TGF-β on the cell surface, increased collagen promoter activity, and resulted in differentiation to a myofibroblastic phenotype, with each of these reduced or reversed with antibody against αvβ5 [226-228]. Thesis data in aggregate from patients with scleroderma showed that up-regulation of αvβ5 expression in fibroblasts increased ECM production and promoted myofibroblastic differentiation through enhanced autocrine TGF-β signaling.

### Potential mechanistic role of inflammation

#### Absence of cellular infiltration does not equate with lack of inflammation

As mentioned previously, recent pathologic descriptions of patients with IPF have emphasized the epithelial, mesenchymal, and ECM abnormalities, while de-emphasizing the possible contributions of inflammation to mechanisms of the disease. Most patients with IPF will manifest a mild to moderate degree of chronic cellular inflammation in the lung pathologically, but it is true that some patients with end-stage lung disease and advanced UIP on biopsy will have minimal evidence of chronic inflammatory cells in the lung parenchyma. Although infiltration with inflammatory cells is an overall common feature of inflammation, the degree of cellular infiltration may vary significantly depending on the particular tissue which is involved. In disease of tendons (tendinopathies), the presence of inflammatory cells is rather limited, due most likely to tissue architecture constraints, but an active inflammatory process is present based on levels of cytokines, growth factors, prostaglandins, and neuropeptides [229,230]. In the spatially confined brain, often considered an “immune-privileged” organ, cellular infiltration following injuries or autoimmune processes is minor, but the brain is fully capable of an active inflammatory response, with cytokines playing key roles [231-233]. Molecular rather than cellular inflammation appears to drive the response to injury in tendons and brain, and the lack of inflammatory cells does not equate with lack of inflammation.

#### Resistance to corticosteroids does not equate with lack of inflammation

An additional concept that should not be equated with lack of inflammation is resistance to corticosteroids. There are several well-established diseases in which inflammation is clearly accepted to be the predominant underlying mechanism, but traditional anti-inflammatory therapy has been poorly or completely ineffective in groups or subgroups of patients [23]. Chronic obstructive pulmonary disease (COPD) is a chronic inflammatory disease of the lungs, with high levels of pro-inflammatory cells and cytokines, but most studies have shown that inhaled or systemic corticosteroids do not significantly reduce cellular or molecular markers of inflammation, and do not improve long-term lung function or survival in patients [234-241]. Asthma is also a prototypical inflammatory lung disease of the airways, and although most patients with asthma respond well to corticosteroids, a subset of patients have steroid-resistant asthma, manifested clinically by a poor response to treatment, and mechanistically by continued expression of pro-inflammatory and profibrotic cytokines in alveolar macrophages and T cells despite exposure to corticosteroids [242-247]. In the autoimmune inflammatory diseases rheumatoid arthritis (RA) and systemic lupus erythematosis (SLE), a subset of patients will respond poorly to corticosteroids, and mononuclear and T cells from these patients showed less inhibition of proliferation and less apoptosis when exposed to corticosteroids [248,249]. Similarly, a proportion of patients with inflammatory bowel disease (IBD) will have a poor or absent response to corticosteroids, and peripheral blood T cells from these patients showed less inhibition of proliferation when exposed to corticosteroids [250,251].

The normal mechanisms by which corticosteroids reduce inflammation have been reviewed in detail [235]. Several mechanisms have been postulated by which patients with inflammatory diseases may manifest resistance to corticosteroid therapy, and these have included abnormalities in bone morphogenetic protein receptor [252], increased phosphorylation and subsequent reduced nuclear translocation of the activated glucocorticoid receptor (GR) [253], reduced expression of the anti-inflammatory protein MKP-1 resulting from increased p38 mitogen-activated protein kinase activity [254], reduced binding affinity of GR for corticosteroids due to nitrosylation of GR by inducible nitric oxide synthase [255], or excessive activation of the transcription factor AP-1 which prevents GR interaction with DNA glucocorticoid response elements [256]. One other particularly noteworthy cause of corticosteroid-resistant inflammation in chronic lung disease results from decreased histone deacetylase activity. Corticosteroids normally reduce inflammation by decreasing transcription of activated inflammatory genes, which occurs at least in part by recruitment of histone deacetylase-2 (HDAC2) [235]. In epithelial cells, inhibition of histone deacetylase activity promoted corticosteroid resistance, and this resistance was reversed by HDAC2 overexpression [257]. Reduced levels of histone deacetylase-2 (HDAC2) have been observed in patients with COPD and refractory asthma [258,259], and one mechanism of reduced deacetylase activity is inactivation of HDAC2 by oxidative stress, which has been demonstrated in epithelial cells, animal models, and patients with COPD [260-262].

Based on all the available mechanistic, animal model, and observational data regarding inflammation and immune mechanisms, it may be a reasonable conclusion that IPF is a chronic inflammatory disease of the lung, but cellular infiltration is minor compared to the substantial changes in pulmonary cytokines, and the absence of clinical improvement in treated patients may be a manifestation of resistance to conventional anti-inflammatory therapies. The fact that oxidative stress has been found capable of promoting steroid-resistant inflammation is certainly a thought provoking concept, given the large amount of data demonstrating high levels of oxidative stress in patients with pulmonary fibrosis, which will be discussed below.

#### Oxidative stress and oxidative signaling

Molecular oxygen is central to aerobic metabolism, is critically required for vertebrates, and must be constantly supplied to ensure survival. However, oxygen is a strong oxidant, and has damaging effects on cells and biologic macromolecules due to formation of reactive oxygen species (ROS). Oxidative stress occurs when there is an imbalance between the generation of ROS and the capacity to detoxify these intermediates, which occurs when generation of ROS is excessive, antioxidant defenses are reduced, or both disturbances occur together. Additionally, extensive evidence suggests that ROS may be also involved with post-translational processing of proteins and intracellular signaling mechanisms in health and disease, including activation or deactivation of signaling factors, regulation of gene expression, and cell differentiation. Over many years, there has been accumulating evidence that oxidative stress and/or oxidative signaling may play a major role in the pathogenesis of pulmonary fibrosis [263-266].

### Overview of reactive oxygen species and antioxidant defenses

Reactive oxygen species are formed by single electron reductions of molecular oxygen, leading to formation of superoxide anion (O_2_^**.–**^), hydrogen peroxide (H_2_O_2_), and the hydroxyl radical (^**.**^OH) [267]. Due to their powerful oxidizing capability, ROS can lead to generation of advanced oxidation molecular products and induce damage to cellular and subcellular structures within the lung, including DNA, proteins, cell membranes, and mitochondria. Reactive oxygen species production may result from several sources in the lung, including the mitochondrial electron transport chain, myeloperoxidase, xanthine oxidase, and NADPH oxidases [263,266].

The enzyme system in the lung that has recently gained the most attention for its ability to produce ROS is the NADPH oxidase family of enzymes, also termed the NOX family of enzymes [268]. The primary function of these enzymes was originally viewed as host defense in phagocytic cells against invading microbes, however, NADPH oxidases have now been found in virtually all tissues, and ROS generated by the NADPH oxidases have been shown to have numerous diverse roles in cellular function [268,269]. Seven isoforms of the NADPH oxidases have been described in mammals, and the isoform NOX4 is the major NADPH oxidase up regulated by TGF-β1 [29,270].

The detrimental effect of ROS is expected to be uniquely most profound in the lung due to its constant exposure to the high oxygen tension of the ambient atmosphere. Consequently, the lung has evolved significant defense mechanisms against cellular damage from reactive oxygen species, which most notably include the family of superoxide dismutases (SOD), the peroxidase catalase, and reduced glutathione [263]. Superoxide dismutase has three isoforms and catalyzes the breakdown of superoxide anion to hydrogen peroxide, and catalase accelerates the breakdown of hydrogen peroxide to water. Reduced glutathione (GSH) is a tripeptide containing the amino acid cysteine, and with its sulfhydryl group, acts as one of the major antioxidants present in the lung [271].

### Observations in humans

Most of the observational data suggest that an oxidant/antioxidant imbalance exists in cells and lung tissue of patients with pulmonary fibrosis. Alveolar inflammatory cells (macrophages, lymphocytes, neutrophils, and eosinophils) and fibroblasts from patients with IPF spontaneously generated higher amounts of ROS than normal controls [147,148]. The amount of myeloperoxidase in the alveolar epithelial lining fluid from patients with IPF was also significantly higher than controls, and within the IPF group, those patients with higher levels of myeloperoxidase had a more rapidly deteriorating clinical course than those with lower levels [147]. Whereas alveolar epithelial lining fluid of normal controls contained high levels of GSH [272], patients with IPF had substantially decreased levels of GSH in epithelial lining fluid, BAL fluid, and BAL cells, and had a decreased ratio of reduced to oxidized glutathione (GSH/GSSG) [273-276]. In patients with IPF, immunoreactivity of gamma-glutamylcysteine synthetase, the rate limiting enzyme in reduced glutathione synthesis, was high in areas of regenerating bronchiolar epithelium, but low in fibrotic areas and fibroblastic foci [277]. Extracellular superoxide dismutase (EC-SOD), the major enzyme responsible for inactivating superoxide anion in the extracellular matrix, was absent by immunohistochemistry in fibrotic areas and fibroblastic foci of patients with IPF [278]. Histopathologic sections of lung tissue from patients with IPF showed high expression of the NADPH oxidase isoform NOX4 in fibroblastic foci and in hyperplastic type II cells [270,279,280]. The constellation of these findings together suggests a significant oxidant/antioxidant imbalance in the lungs of patients with IPF.

### Studies in animal models

The role of antioxidant defenses or effects of antioxidant therapy have been examined animal models of lung injury and fibrosis. The oxidant/antioxidant balance in the lung may be shifted through administration of the antioxidant *n*-acetylcysteine (NAC), a synthetic precursor of reduced glutathione. Intraperitoneal, oral, or aerosolized NAC lead to reduced inflammation (BAL inflammatory cells and acute inflammatory cytokines) and reduced collagen deposition (hydroxyproline and histology) in the bleomycin model [281-283]. Intraperitoneal administration of the antioxidant MnTBAP, a metalloporphyrin, attenuated lung fibrosis in the bleomycin model as assessed by hydroxyproline content, airway dysfunction, and histopathology [284]. Mice who were germline-deficient for extracellular superoxide dismutase (EC-SOD) and received bleomycin demonstrated a significant increase in inflammation and fibrosis (hydroxyproline and histopathology) compared to wild type controls [285], whereas mice that were transgenic for EC-SOD had less acute lung injury and less fibrosis than wild-type controls [286]. Mice that were genetically deficient of Nrf2, a transcription factor which contributes to GSH homeostasis, had increased inflammation, increased epithelial cell death, and increased indices of lung fibrosis (hydroxyproline content, collagen accumulation, histopathologic score) compared with wild-type controls in response to bleomycin challenge [287].

Additional animal models have examined the role of the NADPH oxidase isoform NOX4. In wild-type mice, administration of bleomycin increased protein expression of NOX4 in a time dependent manner, and instillation of small interfering RNA (siRNA) against NOX4 or pharmacologic inhibition of NOX4 reduced histopathologic evidence of fibrosis, lung homogenate hydroxyproline content, and α-SMA production compared with controls [270]. In NOX4-deficient mice, administration of bleomycin resulted in decreased amounts of Smad 2 phosphorylation, α-SMA, procollagen mRNA, total collagen content, and histologic fibrosis compared to controls [280]. Although fibrosis was reduced as a result of NOX4 deficiency, the robust pulmonary inflammation in response to bleomycin was not altered, suggesting that inflammation by itself is not sufficient to drive bleomycin-induced fibrosis. Notably, this decreased fibrosis was associated with less alveolar epithelial cell death compared to controls [280]. These results in combination suggest that oxidative stress may be contributing to both alveolar epithelial cell death and lung fibroblast proliferation in pulmonary fibrosis.

### Mechanistic in vitro studies

Many in vitro studies have examined a variety of oxidant and antioxidant mechanisms in lung cells that involve ROS, glutathione, and cysteine. Alveolar inflammatory cells and alveolar epithelial lining fluid from IPF patients are cytotoxic to primary pulmonary epithelial cells; this cytotoxic effect was reduced by the antioxidant enzyme catalase, and did not occur from exposure to cells and epithelial fluid from normal controls [147]. Lung fibroblasts exposed to GSH had substantially suppressed cellular proliferation, whereas oxidized glutathione disulfide (GSSG) had no suppressive effect on proliferation [288]. Exposure of lung fibroblasts to the oxidized thiol/disulfide couple cysteine/cystine (Cys/CySS) resulted in enhanced fibroblast proliferation, expression of fibronectin, expression of TGF-β1, and expression of Smad3 [289]; these effects did not occur with exposure to reduced Cys/CySS.

Much of the recent mechanistic work regarding oxidative mechanisms in pulmonary fibrosis has centered around the two-way interplay between TGF-β1 and ROS mediated processes. ROS have been shown to activate latent TGF-β1 [290], and TGF-β1 increases production of ROS in human lung fibroblasts [291]. Exposure of epithelial cells and fibroblasts to TGF-β1 decreased levels of GSH, decreased levels of the antioxidant enzymes glutathione reductase and catalase, and increased cell cytoxicity mediated by hydrogen peroxide [292-294]. Inhibiting the synthesis of intracellular GSH content in vitro increased TGF-β1 mediated collagen production [293]. TGF-β1 markedly decreased expression of gamma-glutamylcysteine synthetase [277,292], whereas the acute inflammatory cytokine TNF-alpha caused a mild induction of this enzyme [277]. Lung fibroblasts from patients with IPF that were exposed to TGF-β1 generated H_2_O_2_; these fibroblasts were cytotoxic to pulmonary epithelial cells in co-cultures, and this cytotoxic effect was inhibited by the addition of catalase, or by blockade of H_2_O_2_ generation [295]. TGF-β1has been shown to promote epithelial-mesenchymal transition (EMT), one of the possible sources of increased resident mesenchymal cells in pulmonary fibrosis [12] . The addition of glutathione, NAC, or ROS inhibitors prevented TGF-β1-augmented EMT in alveolar epithelial cells [294], suggesting that oxidative stress mediated by TGF-β1stimulation plays a role in epithelial-mesenchymal transdifferentiation. Additionally, the administration of NAC along with TGF-β1 blocked TGF-β1-augmented collagen gel contraction, expression of α-SMA, and release of fibronectin, vascular endothelial growth factor (VEGF), and collagen [293,296]. The mechanisms by which NAC may affect TGF-β1 signaling appear diverse, as NAC decreased TGF-β1-induced reporter gene activity, decreased phosphorylation of Smad 2/3, reduced active TGF-β1 dimer to an inactive TGF-β1 monomer, and interfered with TGF-β1 receptor signaling [297,298]. These findings suggest that NAC may directly interfere with TGF-β1 signaling and function, in addition to its well-established ability to function as a precursor to glutathione synthesis and as a non-specific scavenger of ROS.

In addition to the well-established observations that TGF-β1 alters cellular oxidant/antioxidant balance, TGF-β1 also specifically induces the expression of the NADPH oxidase isoform NOX4 in numerous mesenchymal cell types. These have included pulmonary artery smooth muscle cells [299,300], cardiac fibroblasts [29], kidney fibroblasts [301], human fetal lung mesenchymal cells [270], and mesenchymal cells from patients with IPF [270]. Furthermore, there has been accumulating evidence that TGF-β1-induced expression of NOX4 is closely tied to myofibroblastic differentiation, and that myofibroblast differentiation is dependent on the generation of ROS by NOX4 [29,270].

Exposure of fibroblasts to TGF-β1 increased expression of NOX4, superoxide anion, α-SMA, and ECM-related proteins (connective tissue growth factor, fibronectin and collagen) [29]. Attenuation of NOX4 expression by siRNA significantly reduced production of superoxide anion, phosphorylation of Smad 2/3, expression of α-SMA, and production of the ECM-related proteins. A similar effect was achieved by inhibiting ROS production with ROS species inhibitors. These findings suggested that TGF-β1 stimulation of NOX4 and generation of ROS were essential for Smad 2/3 phosphorylation and for the full manifestation of the myofibroblast phenotype.

Similarly, in human lung mesenchymal cells, TGF-β1 induced NOX4 expression, induced extracellular release of hydrogen peroxide (H_2_O_2_), upregulated production of α-SMA, and caused collagen gel contraction [270]. Pharmacological inhibition of TGF-β1 receptor signaling or attenuation of Smad-3 expression by specific siRNA inhibited the induction of NOX4 and the release of H_2_O_2_. The TGF-β1-stimulated upregulation of α-SMA and collagen gel contraction was inhibited by catalase and by siRNA-mediated inhibition of NOX4. These findings indicated that H_2_O_2_ production from the upregulation of NOX4 was required for TGF-β1 to induce differentiation to a myofibroblast phenotype.

### Antioxidant therapy in patients with IPF

Administration of *n*-acetylcysteine (NAC) to patients with IPF increased intracellular GSH content and GSH levels in BAL fluid [274,276,292], and the spontaneous oxidative activity of BAL cells decreased following NAC treatment [274]. One multicenter study has been performed looking at antioxidant therapy in patients with IPF [302]. In this study, patients were randomly assigned to receive either prednisone and azathioprine, or the combination of prednisone, azathioprine and NAC. Patients who received NAC had a better preservation of the vital capacity and the diffusing capacity for carbon monoxide at one year, although there was no demonstrated improvement in survival. Despite the extensive amount of data regarding alterations in the oxidant/antioxidant balance in lungs with pulmonary fibrosis, administration of antioxidant therapy to patients has not yet been shown to provide clear-cut clinical benefit [7].

#### Procoagulant milieu in the lung

Essential in the process of wound healing in response to tissue injury are procoagulant signaling mechanisms. Activation of the coagulation cascade by tissue injury contributes to both cessation of bleeding as well as tissue repair. Over the past 15 years, there has been an accumulation of evidence in humans and animal models that imbalances in procoagulant or antifibrinolytic activity are present in the lungs of patients with pulmonary fibrosis. Most of the descriptions of these imbalances have centered on abnormalities of tissue factor, factor Xa, thrombin, or proteinase-activated receptors.

### Overview of coagulation

Coagulation is most often initiated at sites of tissue injury, at which time tissue factor (TF), a transmembrane glycoprotein widely expressed on vascular interstitial cells, becomes exposed to plasma serine proteinases [303,304]. As TF binds to activated factor VIIa in plasma, the extrinsic coagulation pathway begins and continues with the activation of factor X to factor Xa, conversion of prothrombin to thrombin, and conversion of fibrinogen to fibrin, the main component of a mature thrombus. Additional activation of the intrinsic coagulation pathway by thrombin results in the desired effect of sustained hemostasis at sites of tissue injury [305]. The coagulation process is normally opposed by fibrinolysis, the process of cleaving fibrin into degradation products, which is initiated when plasminogen is converted to plasmin by plasminogen activators [305]. The activity of plasminogen activators is balanced by the presence of plasminogen activator inhibitors, and additional naturally occurring anticoagulants include tissue factor pathway inhibitor, which inactivates the TF/VIIa complex, and activated protein C, which inactivates factor V and PAI-1 [305,306].

In addition to promoting hemostasis, thrombin and several of the activated coagulation proteinases bind to members of a family of proteinase-activated receptors (PARs). PARs are transmembrane G-protein coupled receptors that have a unique mechanism of activation in which a portion of the receptor (the tethered ligand) is cleaved by proteolysis and then functions as the ligand for the receptor [305]. Binding of ligand to the major thrombin receptor PAR-1 has been shown to cause multiple downstream effects related broadly to wound healing and repair, and specifically to inflammation, epithelial and mesenchymal cell function, and TGF-β.

### Observations in humans

#### Epidemiologic clues

Epidemiologic observations in humans have suggested a relationship between disorders of the coagulation system and the development of pulmonary fibrosis. In a large cohort of patients in Denmark, patients who were homozygous for a mutation in factor V (factor V Leiden) had abnormalities of several respiratory indices when compared to non-carriers, which included an increase in severe dyspnea, lower forced expiratory volume in one second (FEV_1_) and forced vital capacity (FVC), and increased rate of annual decline in the FEV_1_ and FVC [307]. In another large cohort of patients from Denmark, the relationship between a history of deep venous thrombosis or pulmonary embolism was correlated with the presence of interstitial lung disease or idiopathic interstitial pneumonia [308]. The incidence rates of idiopathic interstitial pneumonia and interstitial lung disease were higher in patients with a history of venous thromboembolism or pulmonary embolism than control patients.

#### Tissue factor (TF)

Elevated levels of TF in BAL fluid have been found in patients with IPF compared with normal controls, and in patients with an acute exacerbation of IPF, BAL levels of TF were markedly elevated [309]. Immunohistochemical analyses revealed large amounts of TF in cuboidal epithelial cells in patients with IPF [309], and in type II pneumocytes of patients with IPF, systemic sclerosis, and cryptogenic organizing pneumonia [310]. Increased TF mRNA and protein were seen in fibroblasts from IPF patients compared with normal lung fibroblasts, and expression of TF assessed by qRT-PCR was up-regulated in fibroblastic foci of these IPF patients [311]. Procoagulant activity was increased in patients with IPF and other forms of diffuse parenchymal lung disease, and this increase in procoagulant activity was TF-dependent [312]. Interestingly, this TF-dependent increase in procoagulant activity correlated with a reduction in lung compliance, which is well-known to occur in patients with IPF.

#### Factor VII and Factor X

Increases in factor VII antigen in BAL fluid and mRNA in alveolar type II epithelial cells were detected in the lungs of patients with IPF compared with normal lung tissue [311]. Similarly, increased expression of factor X mRNA was seen in alveolar septa in the lungs of patients with IPF, whereas there was no significant expression of factor X in normal healthy lung tissue [152].

#### Anti-fibrinolytic activity

Contributing to the balance between procoagulant and anticoagulant mechanisms in the lung are the fibrinolytic plasminogen activators (urokinase-type and tissue-type) and the anti-fibrinolytic plasminogen activator inhibitors (plasminogen activator inhibitor 1 [PAI-1] and plasminogen activator inhibitor 2 [PAI-2]). BAL levels of PAI-1 and PAI-2 were elevated in patients with IPF *vs* control patients, accompanied by no difference in urokinase-type plasminogen activator levels, suggesting an imbalance shifted towards anti-fibrinolytic (or procoagulant) activity in the lungs of patients with IPF [309].

#### PAR-1 and PAR-2

The major thrombin receptor PAR-1 was highly expressed in macrophages and fibroblasts within fibroproliferative foci in patients with IPF, whereas there was only weak staining by immunohistochemistry for PAR-1 in resident alveolar macrophages from normal lung tissue [152,153]. Similarly, in patients with systemic sclerosis, PAR-1 expression by immunohistochemistry was elevated in areas of inflammation and fibroproliferation, but showed only minimal expression in normal control patients [313]. Recently, PAR-1 expression was shown to be up-regulated on activated epithelium within the fibrotic areas of IPF patients, and additionally, was co-expressed in these epithelial cells with chemokine CCL2, also known as monocyte chemotactic protein-1 (MCP-1) [314].

In addition to PAR-1, increased expression of mRNA and protein of PAR-2, which is activated by tissue factor, factor VIIa, and factor Xa, but not by thrombin, was found in lung homogenates and fibroblasts of patients with IPF compared with controls [305,311]. Increased expression of PAR-2 by immunohistochemistry was also seen in type II alveolar epithelial cells and in fibroblastic foci, and tissue factor (TF) co-localized with PAR-2 in these fibroblastic foci [311].

### Studies in animal models

Consistent with an overall procoagulant milieu, administration of bleomycin has been shown to increase expression of procoagulant activity and thrombin in BAL [315], increase PAR-1 expression in lung tissue, alveolar epithelium, and bronchial epithelium [314,316], increase factor X gene expression and factor X mRNA levels [152], and decrease levels of activated protein C [306]. Administration of molecules which favor an anticoagulant milieu has also been performed in the bleomycin model. Aerosolization of heparin or urokinase-type plasminogen activator following bleomycin challenge resulted in reduced soluble collagen and hydroxyproline accumulation, reduced histologic and CT features of fibrosis, and improved lung compliance [317]. Intratracheal administration of activated protein C along with bleomycin resulted in less fibrosis, less hydroxyproline content, and a higher ratio of plasminogen activator to thrombin activity than controls [306]. Administration of a direct thrombin inhibitor along with bleomycin resulted in reduced lung collagen accumulation and connective tissue growth factor (CTGF) mRNA compared with controls [316]. Administration of a direct factor Xa inhibitor along with bleomycin resulted in reduced total lung collagen accumulation compared with bleomycin treatment alone [152]. Adenovirus-mediated gene delivery of tissue factor pathway inhibitor along with bleomycin challenge decreased procoagulant and thrombin activity in BAL, reduced the expression of connective tissue growth factor (CTGF) and TGF-β1 mRNA in BAL, reduced hydroxyproline accumulation in the lungs, and reduced histologic evidence of fibrosis [315].

The effects of gene deletion or insertion using transgene technology have also been studied in the bleomycin model. Administration of bleomycin to mice deficient in PAR-1 resulted in reductions in inflammatory cell recruitment, BAL protein, total lung collagen accumulation, and pulmonary levels of MCP-1 and TGF-β1 compared with controls [153]. Administration of bleomycin to mice transgenic for overexpression of PAI-1 resulted in increased lung hydroxyproline content, whereas lung hydroxyproline content and lung histology in PAI-1 deficient mice were not significantly different than that of mice treated with saline alone [318].

### Mechanistic in vitro studies

Activation of fibroblasts by one or more of the coagulation proteinases via proteinase-activated receptors (PARs) has been studied extensively in vitro over the last fifteen years in relation to the ability to promote fibroblast proliferation and extracellular matrix production. Thrombin is a known mitogen for human lung fibroblasts and inhibits fibroblast apoptosis [311,313,319-323]. Thrombin stimulated mitogen-activated protein (MAP) kinase activation and DNA synthesis in normal mouse fibroblasts, and these responses were absent in fibroblasts from thrombin receptor knockout mice [319]. In addition to its mitogenic effect, thrombin increased production of procollagen in human lung fibroblasts in a dose-dependent manner [324,325]. This increase in procollagen was mimicked by agonist peptides for PAR-1 and abolished by inhibitors of thrombin’s proteolytic activity, suggesting that thrombin was exerting its effects via PAR-1 [324]. Furthermore, thrombin caused collagen gel contraction and stimulated the production of α-SMA in normal human lung fibroblasts in a dose- and time-dependent manner [320], indicating fibroblast differentiation to a myofibroblast phenotype. Both of these effects were mediated via PAR-1 and subsequent protein kinase C-mediated intracellular signaling, and both occurred independently of TGF-β1 [320]. In human fetal and adult lung fibroblasts, thrombin increased production and synthesis of connective tissue disease growth factor (CTGF), a potent fibroblast mitogen [37]. This increased production of CTGF occurred via PAR-1 and was independent of TGF-β1.

Similar to thrombin, factor Xa increased fibroblast proliferation in human and mouse lung fibroblasts [311,321,325], and stimulated procollagen-alpha 1 (I) promoter activity and procollagen production in human and mouse lung fibroblasts [325]. These effects on collagen were mediated via PAR-1, since factor Xa had no effect on procollagen production in PAR-1 deficient mouse lung fibroblasts [325]. Similar to thrombin, factor Xa increased production and synthesis of CTGF mRNA in human lung fibroblasts [37].

Although the above studies indicate that the profibrotic effects of thrombin and factor Xa are mediated predominantly via PAR-1, a similar role for PAR-2 in pulmonary fibrosis has recently been suggested. Exposure of human lung fibroblasts to TGF-β1 increased expression of PAR-2 mRNA and protein [311]. One of the known ligands for PAR-2 is factor VIIa, and factor VIIa exhibited mitogenic effects on human lung fibroblasts [311,321]. This mitogenic effect was mediated via PAR-2 and TF, since it was inhibited by siRNAs against PAR-2 or TF, and was greatly enhanced by the simultaneous overexpression of both receptors [311].

The proliferative and profibrotic effects of the coagulation proteinases on fibroblasts may not only be direct, but may also occur through indirect mechanisms. One described indirect mechanism is activation of PAR-1 on epithelial cells leading to integrin-mediated activation of latent TGF-β1 [152,326]. Another indirect mechanism relates to the described role of epithelial apoptosis in pulmonary fibrosis. Activation of PAR-1 with thrombin or a PAR-1 agonist led to apoptosis of pulmonary epithelial cells, which was reduced by treatment with a PAR-1 inhibitor and in cells with reduced PAR-1 expression via gene silencing [327]. Based on the multiple effects of PAR-1 activation, it is interesting to note that thrombin induced apoptosis in epithelial cells, but inhibited apoptosis in lung fibroblasts. These findings are consistent with one of the major proposed mechanisms for IPF in which pulmonary fibrosis is driven by a combination of apoptosis of epithelial cells and numerical expansion of fibroblasts and myofibroblasts.

### Anticoagulation therapy in patients with IPF

Two trials have been published examining the effects of anticoagulation therapy in patients with IPF. In a study of 20 patients with IPF, the safety and tolerability of inhaled heparin were examined, and demonstrated safety of inhaled heparin and an overall stability in pulmonary function parameters and quality of life scores [328]. In a randomized study of 56 patients, those who received anticoagulation therapy along with prednisolone had an improved overall survival compared with patients treated with prednisolone alone [329], but this study did have limitations which reduced the ability to make generalized conclusions [7]. Despite the extensive amount of data indicating the profibrotic activity of the coagulation proteinases and the procoagulant milieu in the fibrotic lung, administration of anticoagulation therapy to patients has not been shown to provide clear-cut clinical benefit, and is not currently recommended [7].

## Conclusions

Pulmonary fibrosis is a chronic condition of the lungs with characteristic clinical, radiographic, physiologic, and pathologic findings. At the present time, no proven effective therapies exist for pulmonary fibrosis. In some patients, lung injury and subsequent fibrosis occur in which the deposition of excess ECM seems to cease, the impairment in lung function is mild, and the lack of effective therapies does not adversely impact patients. In other patients such as those with IPF, the deposition of excess ECM appears to be progressive, impairment in lung function is severe, and the lack of effective therapies in these patients results in the morbidity and mortality from this disease.

One limitation to advancing our knowledge in this field is that none of the currently accepted animal models of pulmonary fibrosis (bleomycin, radiation, silica, transgenic, viral vectors) accurately mimic human IPF [330,331]. Despite this limitation, an extensive amount of mechanistic work has been performed using these models, with a resultant immense amount of insight gained into epithelial and mesenchymal cell biology of pulmonary fibrosis. In this review, we have attempted to provide an overview of three broad areas which have been explored, and which may be responsible for the observed epithelial, mesenchymal, and ECM abnormalities: inflammation and immune mechanisms, oxidative stress and oxidative signaling, and procoagulant mechanisms. In each of these processes, there is a preponderance of mechanistic, animal model, and human data indicating that disturbances in these mechanisms exist, but at present, pharmacologic targeting of these disturbances in patients has not resulted in improved outcomes. Continued efforts aimed at determining the precise relationship between these disturbances and the development of fibrosis will hopefully lead to effective therapies for patients in the future.

## Abbreviations

α-SMA, Alpha-smooth muscle actin; ECM, Extracellular matrix; EC-SOD, Extracellular superoxide dismutase; EMT, Epithelial mesenchymal transition; GSH, Reduced glutathione; H2O2, Hydrogen peroxide; IPF, Idiopathic pulmonary fibrosis; NAC, n-acetylcysteine; NOX, NADPH oxidase; O2.–, Superoxide anion; ROS, Reactive oxygen species; siRNA, Small interfering RNA; TGF-β, Transforming growth factor-beta; UIP, Usual interstitial pneumonia.

## Competing interests

The authors declare they have no competing interests.

## Authors’ contributions

This manuscript has been seen and approved by all co-authors, and all authors contributed sufficiently to warrant their inclusion on the author list and to take responsibility for the content of this report.
